# Prioritization of antimicrobial resistance surveillance of gram-negative pathogens - a stacked modelling approach

**DOI:** 10.12688/wellcomeopenres.25696.1

**Published:** 2026-02-20

**Authors:** Sneha P Kotian, Rik Oldenkamp, Constance Schultsz

**Affiliations:** 1 Department of Global Health, Amsterdam Institute for Global Health and Development – AIGHD, Amsterdam UMC Locatie AMC, Amsterdam, North Holland, 1105 BP, The Netherlands; 2 Amsterdam Institute for Life & Environment (A-LIFE) Section: Environment & Health, VU University Amsterdam Institute for Environmental Studies, Amsterdam, North Holland, 1081HZ, The Netherlands; 3 Department of Global Health and Department of Medical Microbiology, Amsterdam Institute for Global Health and Development – AIGHD, Amsterdam UMC Location AMC Department of Medical Microbiology, Amsterdam, North Holland, 1105BP, The Netherlands

**Keywords:** antimicrobial resistance, gram-negative bacteria, surveillance prioritisation, stacking, ensemble modelling, gaussian process regression, global health

## Abstract

Antimicrobial resistance in gram-negative pathogens is a growing global health threat, particularly in low- and middle-income countries (LMICs). However, many LMICs lack the laboratory capacity needed for routine surveillance. Affordable, scalable methods are therefore required to guide the prioritisation of surveillance efforts. Although socioeconomic, governance, environmental, and health-system covariates are known to correlate with clinical AMR prevalence, these contextual covariates have not been fully utilised to estimate resistance in settings with limited data. We quantified these associations using a stacked ensemble modelling framework to generate national AMR prevalence estimates for nine priority gram-negative pathogen–antibiotic combinations from 2005 to 2021.

National AMR data were obtained from the *Vivli AMR Register* and *ResistanceMap* and linked with 178 covariates describing socioeconomic conditions, governance indicators, environmental exposures, water and sanitation, and health-system performance. Child models included generalized additive models, gradient boosting machines, random forests, support vector machines, cubist models, and xgboost regressors, whose estimations were combined using spatiotemporal gaussian process regression as a meta-learner. Cross-validated performance was robust for most pathogen–antibiotic combinations, with *R ^2^* values ranging from *0.65 to 0.93.*

Our modelled estimates enable the identification of priority countries for AMR surveillance by jointly considering estimated prevalence, temporal trajectories, and model performance across pathogens. We identified priority countries across multiple pathogen–antibiotic combinations, with estimated prevalence concentrated in regions such as Central Asia, the Middle East and North Africa, Latin America, and sub-Saharan Africa. Many countries within these regions remain unenrolled in the World Health Organization’s Global Antimicrobial Resistance and Use Surveillance System or contribute only sparse data, underscoring persistent gaps in surveillance where resistance is estimated to be high. By integrating AMR data with relevant contextual covariates, our framework fills temporal and geographical gaps. These estimates support evidence-based prioritisation of surveillance strengthening in resource-constrained settings.

## Plain Language summary

Antimicrobial resistance occurs when bacteria no longer respond to antibiotics that previously worked against them. This makes common infections more difficult to treat and increases the risk of severe illness and death. Gram-negative bacteria (such as *Escherichia coli* and *Acinetobacter baumannii etc.)* are of particular concern because they can rapidly acquire resistance to multiple drug classes, including last-line antibiotics. Many low- and middle-income countries lack the laboratory capacity needed to routinely test bacteria for resistance. This creates substantial gaps in the global understanding of where resistance is highest, how it is changing over time, and which settings require urgent support to prevent its spread.

In this study, we combined resistance data from two large international surveillance systems with a comprehensive set of 178 country-level indicators. These indicators describe socioeconomic conditions, governance, environmental exposures, water and sanitation, and health-system performance—factors that previous research has shown to be associated with AMR. Using a suite of statistical and machine-learning models, we quantified how these contextual indicators relate to observed resistance and used these relationships to estimate resistance in countries where data are missing. We generated national estimates for nine priority gram-negative pathogen–antibiotic combinations from 2005 to 2021. For each country, we assessed current resistance levels, recent short-term changes, and longer-term trends. Our findings indicate that several regions—particularly Central Asia, the Middle East and North Africa, Latin America, and sub-Saharan Africa—likely have high or rapidly increasing resistance levels. By integrating laboratory resistance data with these contextual indicators, our modelling approach fills important temporal and geographical gaps in AMR surveillance. The resulting estimates can support governments and global health agencies in identifying where surveillance needs to be established or expanded.

## Introduction

Antimicrobial resistance (AMR) is a global health issue that threatens the effectiveness of antibiotics. ^1^ Gram-negative resistant bacteria are particularly challenging because they possess strong defence mechanisms and can rapidly spread resistant genes. ^2, 3^ These pathogens cause severe infections, including pneumonia, bloodstream infections, and urinary tract infections, and their multidrug-resistant strains leave limited treatment options. ^2, 3^ Recognizing the critical threat posed by AMR, the World Health Organization (WHO) has identified several high-priority pathogens, including *Escherichia coli*, *Klebsiella pneumoniae*, and *Pseudomonas aeruginosa* resistant to third-generation cephalosporins and carbapenems, and *Acinetobacter baumannii* resistant to carbapenems, aminoglycosides, and fluoroquinolones. ^4^ The WHO 2024 report on bacterial priority pathogens emphasizes these combinations to guide the development of new antibiotics, enhance global surveillance, and implement effective infection control measures. ^4^

Efforts to quantify AMR prevalence and burden have progressed significantly over the past decade. The 2019 global burden of AMR was estimated at nearly 1.27 million deaths that were directly attributable to resistant bacterial infections. ^1^ Despite this progress, the global distribution of AMR prevalence remains poorly mapped, with robust surveillance networks largely concentrated in high-income countries (HICs). ^5, 6^ This highlights the urgent need for innovative approaches to fill data gaps, particularly in LMICs, where the burden of resistance is highest and AMR surveillance is underrepresented. ^5, 6^

Predictive models are powerful tools to fill data gaps, allowing estimation of AMR prevalence in regions with limited or no historical surveillance data. Integration of clinical AMR data with relevant socioeconomic, health system, and environmental covariates enables a more comprehensive understanding of both AMR prevalence across countries and the underlying determinants. ^1, 7, 8^ For example, higher AMR prevalence is associated with lower national income levels, reduced access to quality healthcare, and lower educational attainment. ^9, 10^ Poor water, sanitation and hygiene (WASH) systems facilitate the spread of resistant organisms, particularly in densely populated or resource limited settings. ^9, 10^ Additionally, high rates of antibiotic consumption in both humans and livestock, particularly in countries with weak governance, also correlate with the emergence and spread of resistance. ^9, 10^ While previous studies have advanced our understanding, the complexity of AMR requires approaches that can synthesize diverse datasets and offer improved prevalence estimates from comprehensive analyses. ^5^ Ensemble modelling is a powerful approach in this context. ^11, 12^ Unlike traditional single-model approaches, ensemble models integrate predictions from multiple algorithms, leveraging their individual strengths to improve overall performance. ^13^ Additionally, they can integrate diverse data sources and manage complex data types effectively. ^11^ This makes them particularly suited for providing estimates of AMR prevalence. ^6, 14^

This study employs a stacked ensemble model to systematically estimate AMR prevalence at national level, for nine priority gram-negative pathogen-antibiotic combinations over a 17-year period (2005–2021). The model leverages correlations between microbiological, socioeconomic, and environmental covariates to fill critical data gaps. ^7, 8^ Our approach aims to improve accuracy and robustness by integrating predictions from multiple algorithms. By combining national-level AMR data with diverse covariates, we not only can bridge temporal and geographical gaps, but also identify the relative importance of various covariates driving AMR, across countries. Such substantiated estimates should provide a more comprehensive understanding of AMR and support global efforts to prioritise AMR surveillance strategies when resources are limited.

## Methods

### A. Study design

We developed and applied a two-stage spatiotemporal modelling framework to estimate the prevalence of AMR in key gram-negative pathogens at the national level across countries from 2005 to 2021, using sociodemographic, healthcare, environmental, and governance-related covariates. The pathogens included were *Escherichia coli*, *Klebsiella pneumoniae,* and *Pseudomonas aeruginosa,* all resistant to third-generation cephalosporins and carbapenems, and *Acinetobacter baumannii* resistant to carbapenems, aminoglycosides, and fluoroquinolones. Each pathogen–antibiotic combination was modelled independently within the framework, yielding nine distinct spatiotemporal models.

### B. Input data

(I) **Antimicrobial Resistance Data**

AMR data were compiled from two major global surveillance platforms: the ATLAS *(Antimicrobial Testing Leadership and Surveillance)* network, accessed via the Vivli AMR repository, ^15^ and ResistanceMap. ^16^ The integrated dataset included over 5.8 million bacterial isolates, covering 82 countries across multiple world regions. ^15– 17^ The ResistanceMap dataset provided aggregated, country-level resistance rates for invasive bacterial isolates obtained from blood and cerebrospinal fluid specimens, collected from inpatients across all age groups. In contrast, the ATLAS dataset contained isolate-level records with detailed antimicrobial susceptibility profiles, including minimum inhibitory concentration (MIC) values. ATLAS clinical isolates originated from diverse specimen types, including blood, sputum, urine, abscess material, and wound swabs. We classified MIC values from the ATLAS dataset as *susceptible* or *resistant* based on the clinical breakpoints in effect in the country of origin at the time of sampling. Intermediate susceptibility results were reclassified as resistant, consistent with the ResistanceMap methodology. These steps were undertaken to construct a combined dataset with directly comparable antibiograms.

Per pathogen-antibiotic pair, countries were classified as having AMR surveillance if resistance data were available for at least one year between 2016 and 2021. Overall, neither of the two surveillance platforms contained surveillance data for 136 countries. These countries represented approximately 2.24 billion people in 2021. These gaps spanned all income levels, including 46 lower-middle-income countries, 39 upper-middle-income countries, 26 low-income countries, and 25 high-income countries. Regionally, AMR surveillance data were missing in Africa (45 countries), followed by Europe (25), the Americas (24), the Western Pacific (18), the Eastern Mediterranean (15), and South-East Asia (9). In contrast, an average of 53 countries per pathogen-antibiotic combination had reported AMR data, in the European region (28), followed by the Americas (10), the Western Pacific (7), the Eastern Mediterranean (6), and only 2 each in the African and South-east Asian regions.

(II) **Covariate Data**

We conducted a literature review to identify covariates commonly used in prior antimicrobial resistance and global health modelling studies. *(Supplementary Appendix)* These covariates were subsequently grouped into thematic domains encompassing socioeconomic factors, health-system capacity and financing, governance, environmental exposures, and water, sanitation, and hygiene (WASH). Covariates identified through this process were then extracted from publicly available global datasets and prepared for modelling. We included 1,897 covariates from the World Bank dataset and the WHO Global Health Repository, encompassing data on health, nutrition, antimicrobial consumption, population, and development. ^18, 19^ The dataset covered 242 geographical locations, including countries and territories, with records spanning 2005 to 2021.

We implemented a multi-step filtering process to ensure data quality. First, only those covariates were included that covered at least 80% of country-year combinations. ^20^ Duplicate covariates across the two sources were identified and removed. Missing data were imputed using the ‘missForest’ function from the R package ‘missForest’. ^21^ This function applies a non-parametric method utilising random forests to iteratively predict missing data by understanding complex interactions and nonlinear relationships in datasets, and is particularly effective for time-series and continuous data. ^21, 22^ After imputation, all covariates were standardized to ensure comparability and to prevent scale-related biases in the modelling process. 178 covariates were retained as input variables for subsequent analysis.

### C. Modelling framework

The AMR and covariate datasets were merged by country and year. All country-year combinations for which surveillance data were lacking but covariate data were available, were excluded from model training and testing, but were included in the prediction phase to generate modelled estimates. Complete records, i.e., those containing data on both AMR surveillance and covariates, were used for model training and testing.

To enhance statistical reliability and minimise the influence of small sample sizes, we excluded country–year observations with fewer than 30 isolates. ^23^ We performed a stratified 75%–25% train–test split for each pathogen–antibiotic dataset, to achieve proportional representation of WHO regions and income groups. To check for representativeness and minimise sampling bias, the mean resistance prevalence was compared between training and test data using a two-sample t-test, and income group and region distributions were compared between the datasets using Chi-square tests. The Chi-square test results showed no significant differences in the distribution of income groups or regions between the training and test datasets. For each pathogen–antibiotic combination, the two-sample t-test did not provide evidence for a difference in mean AMR prevalence between training and test sets (all p-values >0.05).

A stacked ensemble framework was employed to harness the combined strengths of multiple machine learning child models and enhance predictive performance. The machine learning model types that were employed as child models included generalized additive models *(GAM)*, gradient boosting machine *(GBM)*, random forest *(RF)*, cubist regression models, support vector machine *(SVM)*, and xgboost regression models. Each child model was trained using five-fold cross-validation to prevent overfitting and improve generalizability. We selected these child models because of their unique and complementary capabilities. GAMs contribute by capturing smooth, non-linear associations between resistance and covariates. ^24^ Tree-based learners such as *RF, GBM*, and *xgboost* are particularly effective for handling complex interactions and non-linear thresholds. ^22, 25^ Cubist offers rule-based regression that performs well in estimation tasks. ^26^ *SVMs*, by contrast, are effective in identifying subtle, high-dimensional patterns, making them well-suited to datasets with diverse covariates. ^27^ By combining these diverse learners, the ensemble balances interpretability, flexibility, and predictive accuracy, thereby reducing reliance on any single algorithmic assumption.

The outputs from the child models were used as input features for a spatio-temporal gaussian process regression (ST-GPR) meta-model. ST-GPR was chosen as the meta-model for its ability to capture complex, non-linear relationships. ^28^ ST-GPR operates within a probabilistic framework, modelling a distribution of outcomes rather than producing single-point estimates. Furthermore, GPR naturally accounts for spatial and temporal correlations, enabling more informed estimates by leveraging similarities across neighbouring regions and time points. ^28^

All models, including the ST-GPR meta-model, were implemented in R (version 4.5.0) using the packages *mgcv* (v 1.9-3), ^29^ *gbm* (v 2.1-8), ^30^ *randomForest* (v 4.7-1), ^31^ c *ubist* (v 0.3-0), ^32^ *xgboost* (v 1.7.5), ^33^ *caret* (v 7.0-1), ^34^ and *INLA* (v 22.12.16). ^35^

### D. Model performance

For each pathogen-antibiotic combination, child model performance was evaluated using root mean squared error (RMSE) and coefficient of determination (R ^2^) for both in-sample (training; R ^2^_is_) and out-of-sample (testing; R ^2^_oos_) data. The best-performing models were then used as inputs for the meta-model, enabling it to combine their strengths for improved final estimations.

For each child model, the relative importance of input covariates was quantified using model-specific variable importance functions (e.g., *varImp* in *caret*). ^34^ The importance scores were then averaged across all child models to obtain a single ranked estimate of each covariate’s contribution to explaining variation in resistance.

The ST-GPR meta-model was trained separately for each pathogen-antibiotic combination using estimations from the best child models along with spatial and temporal covariates, using 5-fold cross-validation. Performance was assessed on the out-of-sample testing dataset through 1,000 bootstrap resamples, calculating the R ^2^_oos_, nRMSE (normalized root mean square error) and RMSE to quantify predictive accuracy. nRMSE provides a scale-independent measure of model prediction accuracy. Mean values and 95% uncertainty intervals were reported *(Supplementary Appendix)*.

### E. Model application – AMR prioritisation indicators

After testing and validation, the final stacked ensemble model was applied to all countries lacking AMR surveillance data for each pathogen–antibiotic combination. Modelled estimates were produced only where surveillance was absent. Three indicators for prioritization were derived for each pathogen–antibiotic pair: absolute AMR prevalence in 2021, short-term trends measured as the absolute change in prevalence from 2016 to 2021, and long-term trends based on the absolute change from 2005 to 2021. These metrics allow us to identify countries with the highest resistance levels in 2021 as well those experiencing most rapid short-term and long-term increases, thereby informing global priorities for AMR surveillance.

## Results

### A. Model performance

**Figure 1.**
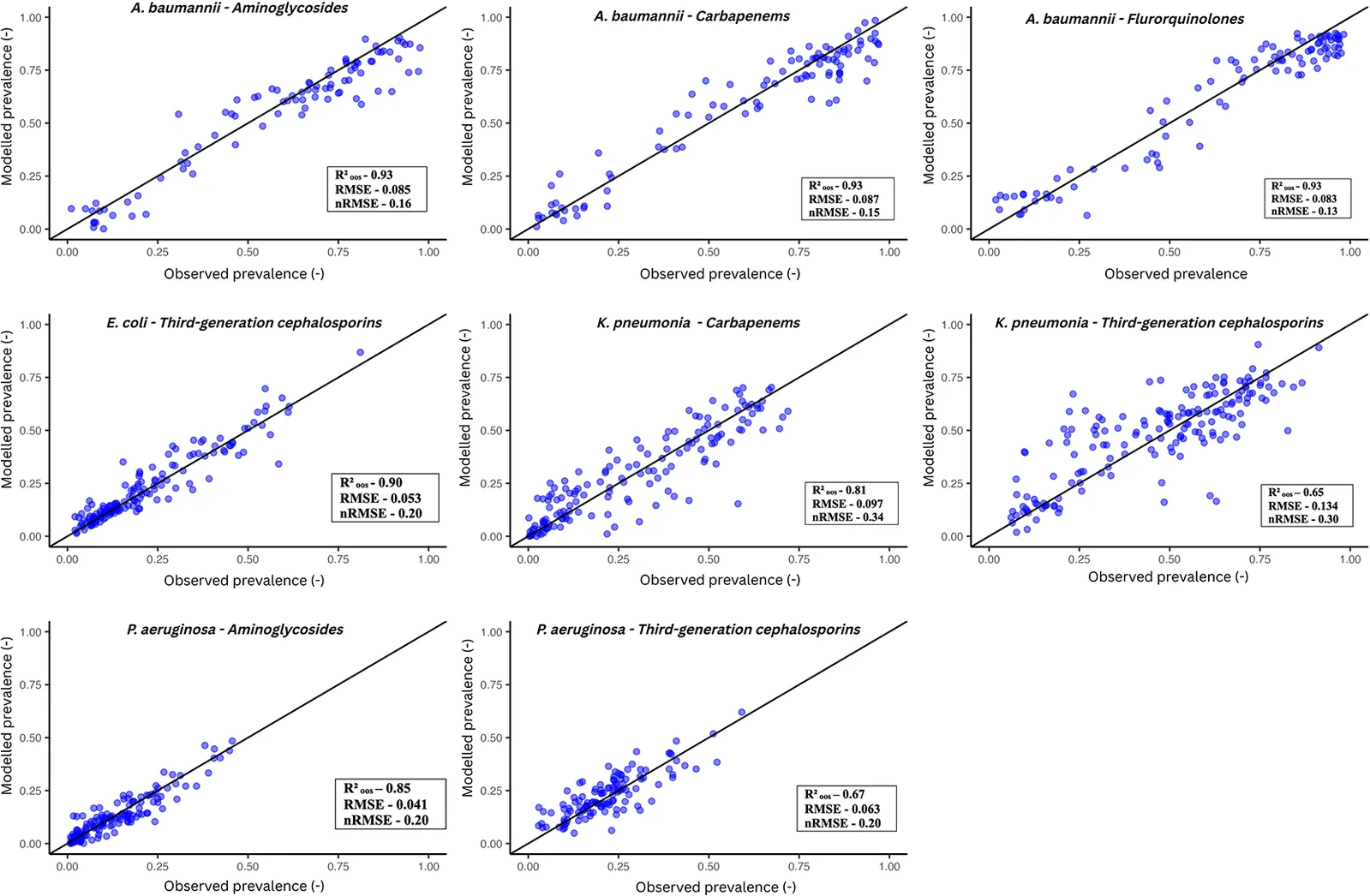
Comparison of observed and estimated resistance prevalence for eight out of nine priority pathogens. R ^2^_oos_: out- of- sample coefficient of determination; RMSE: root mean square error; nRMSE: normalised RMSE. Solid line represents the 1:1 line.

Performance of the meta-models was assessed on the out-of-sample testing datasets using 1,000 bootstrap resamples. Per pathogen-antibiotic combination, the predictive accuracy was summarised using the out-of-sample coefficient of determination (R ^2^_oos_), RMSE, and normalised RMSE (nRMSE), as shown in Figure 1. The predictive performance of the meta-model was generally high for most pathogen–antibiotic combinations. R ^2^_oos_ values ranged from 0.65 to 0.93, with the highest performance observed for *A. baumannii* resistant against aminoglycosides, carbapenems, and fluoroquinolones (R ^2^_oos_- 0.93). For *E. coli* resistant to third-generation cephalosporins, model fit was similarly high (R ^2^_oos_ - 0.90). Performance was slightly lower for *K. pneumoniae* and *P. aeruginosa*, with R ^2^_oos_ ranging from 0.65 to 0.85. Prediction errors as evaluated by RMSE and nRMSE values were correspondingly small for high-performing pathogen-antibiotic combinations. We excluded *E. coli* resistant to carbapenems from our final results. The observed resistance prevalence was (near) zero in many countries, leading to limited variation for the model to capture.

### B. AMR prioritisation indicators

We selected *carbapenem-resistant A. baumannii (*CRAB) and third-generation cephalosporin-resistant *E. coli* (3GCR-EC) to illustrate our main findings and discuss the implications for AMR surveillance prioritisation. A full summary for all pathogen-antibiotic combinations is provided in the *Supplementary Appendix.* CRAB is a major cause of hospital-acquired infections, notable for its resistance to last-line antibiotics and persistence in healthcare environments. ^4^ 3GCR-EC is prevalent in both community and hospital settings, with resistance that severely limits treatment options. ^4^ Both are classified by WHO as critical priority pathogens due to their resistance profiles and widespread impact. ^4^ Their inclusion further aligned with the study by Murray et al. ^1^ which highlighted the accelerating global burden of carbapenem-resistant gram-negative infections and projected substantial increases in AMR-related deaths by 2050, even with the introduction of new antibiotics. AMR prioritisation indicators, as described above, were generated for CRAB and 3GCR-EC for 136 and 132 countries lacking surveillance data, respectively.

*Carbapenem-Resistant A. baumannii (CRAB)*

**Figure 2.**
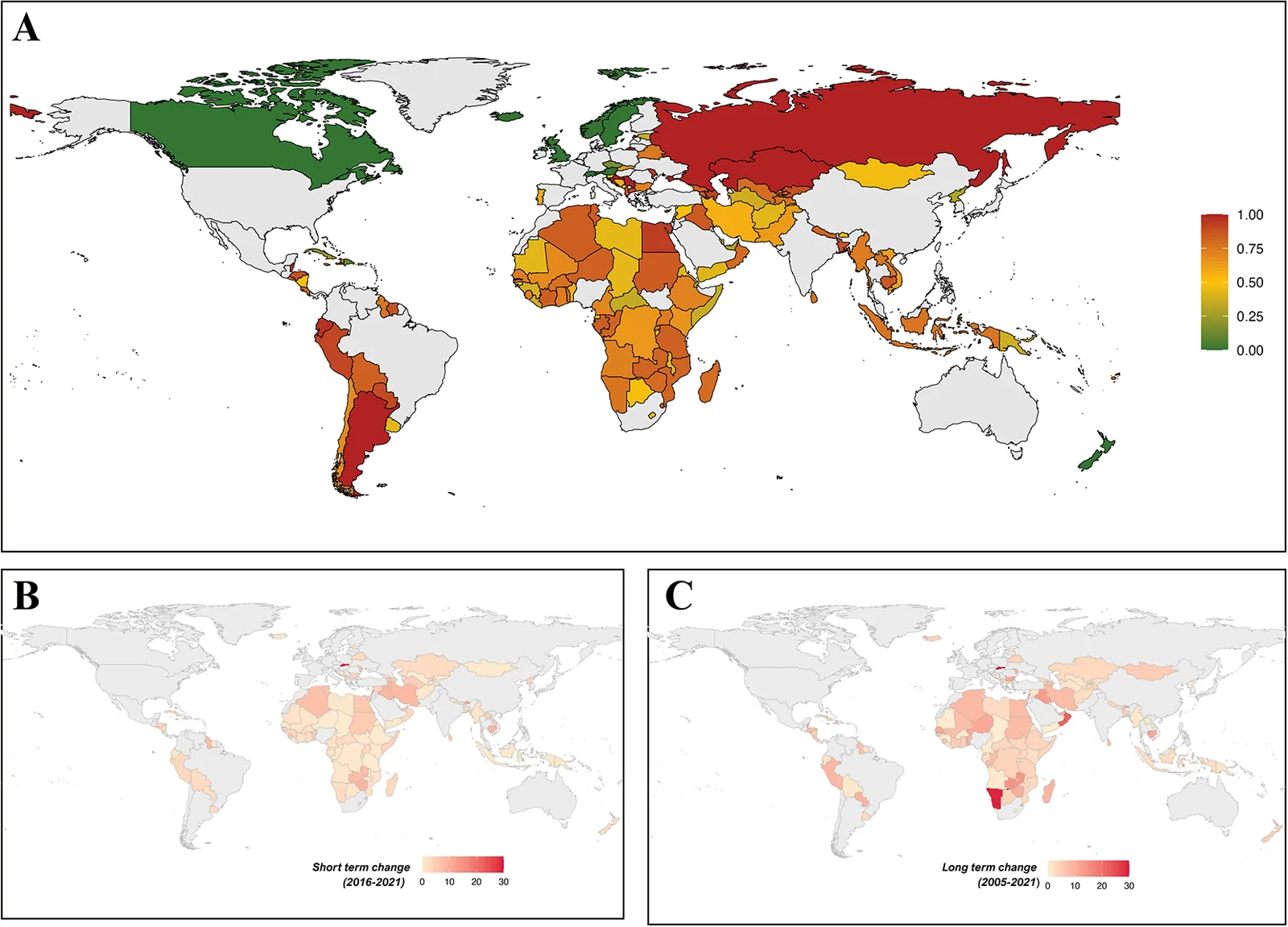
Modelled estimates of prevalence and temporal changes of carbapenem-resistance in *A. baumannii* (CRAB). Results are shown as modelled estimates of national-level prevalence in 2021 (A). Modelled estimates are included for countries lacking surveillance data. Maps display countries colour-coded by the magnitude of absolute change in prevalence, with short-term % change measured from 2016 to 2021 (B) and long-term % change from 2005 to 2021 (C). As observed changes in prevalence did not exceed 30 percentage points, the figures display changes up to a maximum of 30% for visual clarity.

In 2021, the estimated prevalence of carbapenem resistance among *Acinetobacter baumannii* isolates was modelled to be high, with values above 90% in several countries, as shown in Figure 2 *A.* Kazakhstan had the highest estimated prevalence, in the European region. Similarly, in the Americas, the upper-middle income countries Ecuador and Belize had modelled carbapenem resistance among *A. baumannii* isolates of approximately 96% and 95%, respectively. In the Eastern Mediterranean region, Egypt was estimated at around 94%, while in the European region, Azerbaijan had an estimated resistance prevalence of approximately 93%. Other countries including Moldova, Peru, North Macedonia, Paraguay, Albania, Bangladesh, and Suriname also had modelled estimates above 85–90% in 2021.

Short-term patterns based on our modelled estimates indicate that many countries experienced rapid increases in carbapenem resistance in *A. baumannii* between 2018 and 2021 ( Figure 2 *B*). For example, Vanuatu and Namibia each had estimated substantial rises in in the proportion of resistant *A. baumannii* over this period. Cyprus also experienced a marked increase, while conflict-affected Syria had steeply climbing modelled resistance levels. Additional countries with estimated short-term surges included Oman, Cape Verde, and several sub-Saharan African nations such as Senegal, Niger, and Togo.

Long-term trajectories from our modelled estimates suggest that several countries have undergone substantial increases in carbapenem resistance in *A. baumannii* over the past decade ( Figure 2 *C*). We estimate that in the European Region, Slovakia experienced one of the most pronounced rises, shifting from moderate to much higher modelled resistance levels. In the African region, Mauritius, and in the Americas, the Bahamas, also had estimated notable modelled long-term increases.

Third-generation cephalosporin-resistant *E. coli* (3GCR-EC).

**Figure 3.**
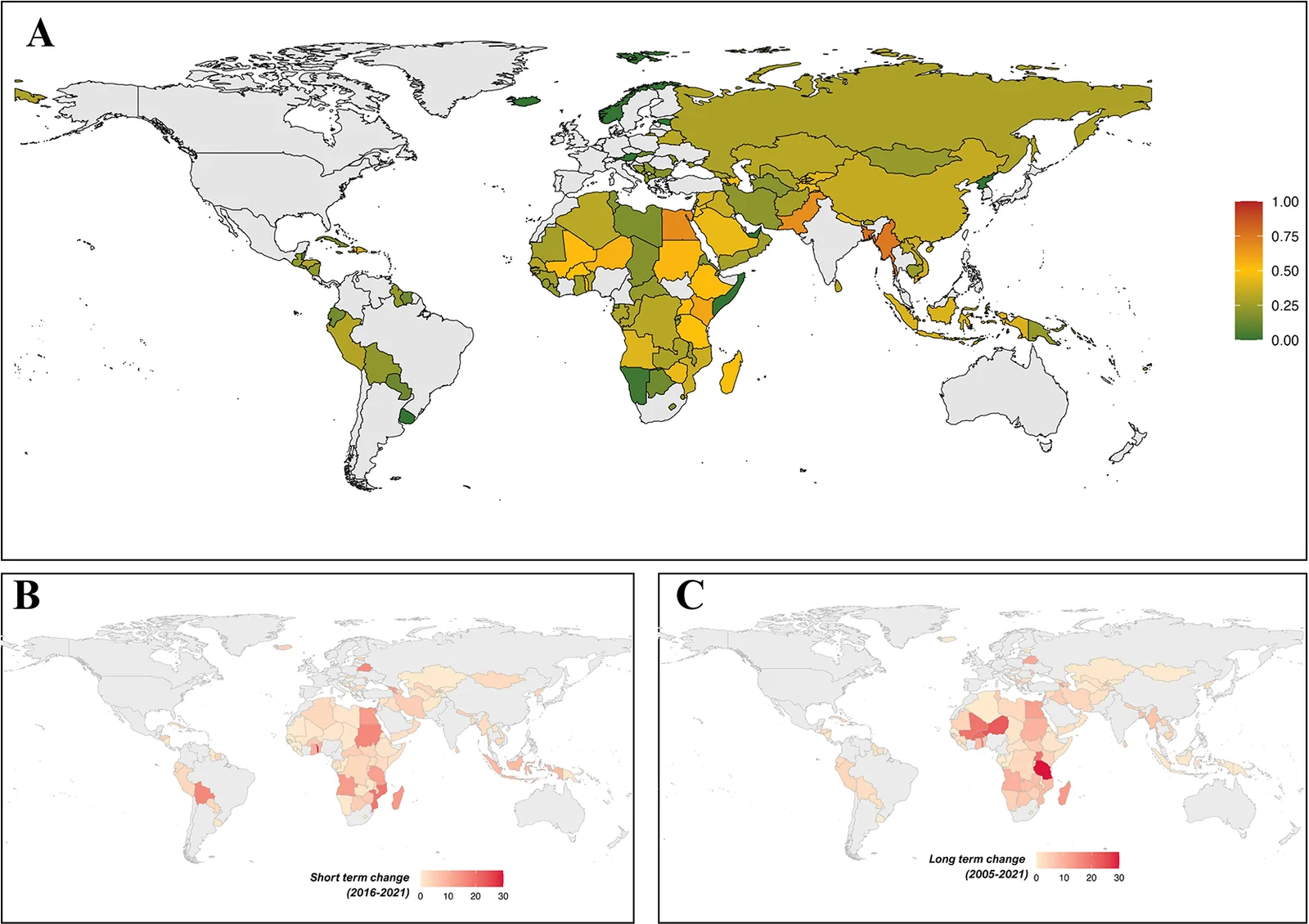
Modelled estimates of prevalence and temporal changes in third-generation cephalosporin-resistance in *E. coli* (3GCR-EC). Results are shown as modelled estimates of national-level prevalence in 2021 (A). Modelled estimates are included for countries lacking surveillance data. Maps display countries colour-coded by the magnitude of absolute change in prevalence, with short-term change measured from 2016 to 2021 (B) and long-term change from 2005 to 2021 (C). As observed changes in prevalence did not exceed 30 percentage points, the figures display changes up to a maximum of 30% for visual clarity.

Our modelled estimates indicate substantial and widely distributed resistance against 3GC in *E. coli* by 2021, with particularly high levels in South-East Asia and sub-Saharan Africa, as shown in Figure 3 *A.* Modelled estimates were highest in Bangladesh (79.2%), followed by Myanmar (75.8%), Egypt (73.4%), and Tanzania (66.3%). Several African countries also exceeded 50%, including Kenya, Niger, Mali, Sudan, Burkina Faso, Ethiopia, and Uganda. Other countries showed slightly lower but still concerning levels, such as Nepal and Indonesia, alongside additional African (e.g., Togo, Madagascar, Zimbabwe, Angola) and Middle Eastern countries. (e.g., Syria, Tunisia, Djibouti, Mauritania).

As per our modelled estimates, some of the most striking recent increases in third-generation cephalosporin-resistant *E. coli* were concentrated in parts of East and West Africa *(* Figure 3 *B).* Countries such as Tanzania, Niger, Mali, and Uganda showed particularly sharp rises 3GC resistance estimates. Additional spikes were also evident in Burkina Faso, São Tomé and Príncipe, and Eswatini. By contrast, South and Southeast Asian countries such as Bangladesh and Myanmar showed more gradual but still concerning upward trajectories, while certain parts of Europe, including Cyprus, displayed only modest increases. For the long-term trends, our modelled estimates indicate that many countries have followed flat-to-rising trajectories, with particularly steady growth visible in parts of West Africa and the eastern Mediterranean *(* Figure 3 *C).*

### C. Role of covariates

While all selected covariates were included in the modelling process, we present the top 20 covariates with the highest importance scores *(Supplementary Appendix, Table no S3-S10).* The most influential covariates spanned multiple domains: health system capacity and financing, governance, socioeconomic development, environment and WASH, agricultural practices, climate, nutrition and chronic diseases. These scores reflect the relative importance of each covariate within the models’ estimation framework but do not signify causality. The relative percentages reported below for each covariate domain reflect their aggregate contribution at the domain level, while the numbers in parentheses indicate the covariate-level importance within each domain.

For CRAB, the largest contribution arose from governance-related covariates (30%), with control of corruption (0.90) as the top contributor, followed by government effectiveness (0.44), voice and accountability (0.16), and rule of law (0.16). Health system financing covariates (25%) also carried substantial weight, with health expenditure per capita (0·54) and health expenditure as a percentage of GDP (0.48) among the leading covariates. Socioeconomic and demographic covariates (25%) contributed through GDP per capita (0.48), net national income (0.40), and crude death rate (0.34). Nutritional and chronic disease covariates (10%), including anaemia in women (0.32) and mortality from non-communicable diseases (0.21), also featured prominently. Finally, environmental and geographical covariates (10%), such as permanent cropland (0.18) were among the higher-ranking contributors.

For 3GCR-EC, socioeconomic and demographic covariates were most important (28%), including adjusted net national income (0.45), GDP per capita (0.41), and urban population growth (0.28). Health system financing and capacity covariates (25%) were similarly influential, led by current health expenditure as a percentage of GDP (0.89) and health expenditure per capita (0.39). Governance-related covariates (22%) also ranked high, particularly political stability (0.62), control of corruption (0.54), and voice and accountability (0.49). WASH-related covariates (12%) contributed through access to clean fuels for cooking (0.52) and basic sanitation services (0.31). Nutritional risk factor covariates (10%), including anaemia in women of reproductive age (0.33), and environmental and agricultural covariates (8%), such as aquaculture production (0.27), made smaller contributions.

## Discussion

Our study provides country-level prevalence estimates of AMR in key gram-negative pathogens over a seventeen-year period (2005-2021). We developed and applied a stacked ensemble framework, which combines multiple machine learning models, and synthesized their outputs using a ST-GPR meta-model. This builds on and advances previous work, ^8^ in which beta-binomial principal component regression models were applied in a similar framework. By combining a flexible ensemble structure with ST-GPR smoothing to stabilise predictions, the current model improves the precision of earlier AMR estimates. Moreover, it operationalises and strengthens the earlier methodological framework by strategically integrating epidemiological and socio-demographic covariates. Together, these enhancements allow the stacked ensemble framework to offer improved practical utility for supporting AMR surveillance and priority-setting.

Our final meta-model achieved robust performance metrics across eight pathogen–antibiotic combinations ( Figure 1). These metrics are broadly consistent with results reported in large-scale global modelling efforts addressing AMR. The study by Naghavi et al., ^6^ employed a similar stacked ensemble with ST-GPR to estimate the burden of AMR, with a performance comparable to our model. Regional analyses using ensemble-based modelling have recently been published for several WHO regions, including the African region, the European region, the Eastern Mediterranean region, and the Region of the Americas. ^14, 36– 38^ In a subnational modelling study spanning 75 countries, Browne et al., ^39^ estimated the prevalence of bacterial resistance and reported R ^2^ values of 0.69–0.78 and RMSE values of 0.17–0.18. Taken together, these results support ensemble modelling as a useful approach for generating reliable AMR estimates in data limited settings.

Our modelled estimates of third-generation cephalosporin resistance among *E. coli* isolates and carbapenem resistance among *A. baumannii* isolates reveal gradients across countries, ranging from long-standing high-resistance settings to others where resistance remains lower but shows signs of recent increase. To contextualise these patterns, we describe countries within their respective geographical regions—Central Asia and Europe, the Middle East and North Africa (MENA), sub-Saharan Africa, Latin America, and the Asia-Pacific Islands. When compared with our previous work, ^8^ the broad global distribution of high prevalence of resistance remains consistent, particularly in South Asia, the Middle East, Latin America, and parts of Africa and Eastern Europe.

### Central Asia and Europe

Kazakhstan showed the highest estimated prevalence of carbapenem resistance in *A. baumannii* in the region, exceeding 90% in 2021. This aligns with findings from a multi-centre national study conducted in Kazakhstan, which reported the prevalence of carbapenem resistance ranging from 78% to 81% among *A. baumannii* isolates between 2011 and 2019. ^40^ Comparable patterns have been documented across Southeastern Europe, with carbapenem resistance exceeding 85–100% in intensive care settings in Bulgaria and Moldova respectively. ^41, 42^ In contrast, Slovakia presents a different profile: while current prevalence remained moderate, our model indicated a marked long-term increase, in line with a hospital-based study showing progressive emergence of drug-resistant *A. baumannii.* ^43^ Our estimates showed moderate prevalence and slow-to-flat trends in this region for 3GCR-EC. These results align with the WHO European region analysis by Ikuta KS et al. ^37^ This study showed that the prevalence of resistance to 3GC in EC was notably higher in central and eastern Europe than in northern or western Europe, mirroring the gradient captured by our model.

### Middle East and North Africa (MENA)

Our modelled estimates identified Egypt as a key regional hotspot for both pathogens. For carbapenem resistance in *A. baumannii*, estimated prevalence exceeded 90%, consistent with a hospital-based study reporting resistance levels of 94–98% and frequent detection of carbapenemase-producing isolates. ^44^ For resistance to 3GC in EC, Egypt also exhibited high prevalence, in line with regional evidence showing sustained third-generation cephalosporin resistance across north Africa and south Asia since the early 2000s. ^45^

Comparable trends have been reported from Tunisia, where Dziri et al., ^46^ described over a decade of sustained circulation of carbapenemase-producing gram-negative bacteria along with rising prevalence levels. Oman showed a notable short-term rise in prevalence of carbapenem resistance in *A. baumannii* between 2018 and 2021, consistent with observations by Sannathimmappa et al., ^47^ who reported carbapenem resistance rates of 70–72% among *A. baumannii* isolates from Omani hospitals. Cyprus demonstrated a similar short-term increase but with sparse longitudinal data. In Syria, both pathogens exhibited rising prevalence. A clinical study from Syria confirmed the circulation of highly resistant *A. baumannii*, and additional evidence showed increasing third generation cephalosporin resistance among *E. coli* isolates. ^45, 48^

### Sub-Saharan Africa (SSA)

Across SSA, our modelled estimates reveal concerning upward trends in resistance prevalence for both carbapenem resistance in *A. baumannii* and third-generation cephalosporin resistance in *E.coli.* For carbapenem resistance in *A. baumannii,* rapid short-term increases were identified in Namibia, Senegal, Togo, and Niger, while Zimbabwe and Uganda showed rising longer-term trajectories. These findings are consistent with data from Zimbabwe, where Mhondoro et al. documented a progressive rise in resistance among *A. baumannii* and *Pseudomonas* species between 2012 and 2017. Similar evidence from Uganda showed early emergence of carbapenem resistance in *A. baumannii* in tertiary hospitals, as reported by Kateete et al. for the period 2007–2009. ^49, 50^ A recent meta-analysis by Arowolo et al., ^51^ estimated the pooled prevalence of carbapenem resistance among *Acinetobacter baumannii* clinical isolates in sub-Saharan Africa at 20% (95% CI 4–43%), highlighting substantial heterogeneity across studies.

For third generation cephalosporin resistance among *E. coli* isolates, our model revealed rapid short-term increases in Tanzania, Niger, and Mali, with sustained long-term growth in Uganda, Sudan, and Ethiopia. These findings align with the systematic review by Lester et al., ^52^ which documented high prevalence and increasing temporal trends of third generation cephalosporin resistance in *E. coli* across SSA. In Sudan, rising ceftriaxone resistance among *E. coli* isolates has been reported in recent years, corroborating the accelerating trends captured in our model. ^53^ Country-specific evidence from Uganda and Ethiopia further supports these patterns. In Uganda, Mayito et al., ^54^ reported *E. coli* resistance exceeding 70% in tertiary hospitals between 2020 and 2023, consistent with our estimates placing the country in the highest quartiles for both short- and long-term growth. Similarly, in Ethiopia, Abdeta et al., ^55^ documented rising carbapenem resistance among *A. baumannii* and *Pseudomonas* species, while Gashe et al., ^56^ reported ceftriaxone resistance above 50% in *E. coli* isolates. Both these findings were in close agreement with our long-term trends. Collectively, these patterns identify SSA as an emerging acceleration zone where surveillance and laboratory capacity currently remain insufficient.

### Latin America and the Caribbean

In Latin America, our estimates identified Ecuador, Peru, Belize, Paraguay, and Suriname as high prevalence countries for carbapenem resistance in *A. baumannii.* These findings are consistent with a hospital-based study from Peru, which reported prevalence of carbapenem resistance exceeding 90% among *A. baumannii* isolates from tertiary hospitals. ^57^ Similarly, another study from Ecuador showed increasing resistance prevalence among gram-negative bacilli in clinical isolates from healthcare facilities across Ecuador. ^58^

### Asia and Pacific Islands

Across the Asia–Pacific region, several Southeast Asian countries showed high estimated prevalence of both carbapenem resistance in *A. baumannii* and third generation cephalosporin resistance in *E. coli.* Bangladesh and Myanmar exhibited high prevalence of third generation cephalosporin resistance in *E. coli,* consistent with multiple hospital-based studies. ^59, 60^ For carbapenem resistance in *A. baumannii*, Bangladesh also featured prominently, consistent with regional meta-analyses estimating mean prevalence of 56% across Asia, reaching >90% in certain countries. ^61^ In the Pacific, studies remain limited demonstrating the need for modelling studies to provide estimates. Fiji and Vanuatu showed notable short-term increase in carbapenem resistance in *A. baumannii.* A study from French Pacific Territories documented local carbapenem resistance in *A. baumannii* prevalence around 25%, suggesting under-detection in island settings. ^62^

Taken together, our modelled estimates reveal pronounced geographical heterogeneity in resistance levels and trajectories across both pathogens, that are in line with the limited available data published. Some countries, such as Kazakhstan, Egypt, Bangladesh, and several in Latin America, appear to have reached persistently high prevalence, indicating that resistance is well established and unlikely to decline without major intervention. Others, including Oman, Namibia, Niger, and Tanzania, show sharp recent increases, suggesting that resistance is expanding rapidly and may soon become entrenched. A third group, including Uganda, Sudan, Ethiopia, and Slovakia, exhibits slower but steady long-term increases, reflecting gradual increase over time rather than stabilization.

### Role of covariates

Across child models, socioeconomic and demographic covariates ranked highest in variable-importance for 3GCR-EC. Covariates such as income, education, and urban growth were highlighted, a pattern consistent with evidence that overcrowding and socioeconomic barriers amplify exposure to resistant pathogens. ^63^ Densely populated settings may amplify transmission of enteric bacteria; notably, ecological analyses have linked increases in population density with higher resistance prevalence in pathogens like *E. coli.* ^64^ In addition, covariates from the WASH domain, such as use of basic sanitation services and reliance on unsafe water sources, also featured as important covariates. Poor sanitation and contaminated water enable the transmission of resistant organisms such as *E. coli* in the community, leading to more infections that require antibiotic treatment and thereby increasing selective pressure. ^64^ Several covariates reflecting health system strength and access, including healthcare expenditure, hospital infrastructure, and laboratory capacity, carried substantial weight in our model for CRAB. These covariates likely represent how effectively infections are managed and antibiotics are utilized within individual countries. Health system capacity was also identified as an important covariate domain for CRAB, reflecting its hospital ecology and dependence on infection-prevention and stewardship practices. Previous studies have shown that CRAB transmission is largely driven by failures in infection-control infrastructure and antibiotic stewardship, particularly in resource-limited hospitals. ^10, 61, 65^ Its ability to persist on surfaces and within hospital water systems allows sustained transmission when diagnostic capacity, device reprocessing, and isolation practices are weak. ^66^

Governance was the most influential covariate domain for CRAB, with control of corruption the single strongest contributor. Covariates such as regulatory quality, control of corruption, and government effectiveness, capture a country’s ability to implement effective AMR control strategies. A modelling study in both high-income and low-income countries suggested that governance indicators (e.g., the extent of corruption) explain variation in AMR better than antibiotic consumption alone. ^67^ Weak governance can translate to inadequate regulation of antibiotic sales, ineffective hospital oversight, and limited accountability for AMR action plans. ^68, 69^ In contrast, countries with strong institutions are more likely to enforce prescription-only antibiotic use, invest in public health, and curtail inappropriate practices. ^68, 69^ An assessment of 15 African national AMR action plans found major gaps in transparency, accountability, and implementation, underscoring how weak governance undermines resistance control—by contrast, countries such as Sweden, Norway, Iceland demonstrate how strong institutions and clear accountability mechanisms translate into lower resistance levels and more effective stewardship. ^69, 70^

Although smaller in relative contribution, several environmental covariates, often not directly related to health, were also highlighted. Covariates such as cropland use and aquaculture production might act as proxies for industrialisation and agricultural antibiotic use, providing a ‘One Health’ perspective on AMR transmission. ^71, 72^

Nutritional and chronic disease covariates, including anemia, obesity, and diabetes, also emerged as important covariates. These reflect underlying vulnerabilities that increase infection risk and antibiotic use. For example, in sub-Saharan African settings, severe anemia is strongly associated with increased risk of community-acquired bacteraemia. ^73^ Such bloodstream infections frequently involve gram-negative pathogens, indicating that areas with a high anemia burden face elevated risk of gram-negative infections and subsequent antibiotic use. ^73^ Patients with chronic diseases often have immune dysregulation and frequent healthcare contact, which increases opportunities for infections and antibiotic treatments. ^74^ These examples highlight how extremes of malnutrition and rising non-communicable disease prevalence converge with AMR and threaten vulnerable populations.

### Prioritisation of AMR surveillance

Our study allows the identification of priority countries for AMR surveillance, by jointly considering model performance, resistance trajectories, GLASS enrolment, covariate profiles and the clinical relevance of key gram-negative pathogens.

First, we observed a strong cross-pathogen convergence: many countries surfaced as priorities for both carbapenem-resistant *A. baumannii* and third-generation cephalosporin-resistant *E. coli.* as well as for the other pathogen-antibiotic combinations. When countries emerge as high priority across multiple pathogens, it might indicate that resistance dynamics are shaped by shared structural drivers such as weak infection control, high community transmission, and inadequate stewardship. It also emphasizes that national surveillance gaps are not isolated problems but reflect broader weaknesses in microbiological capacity and health system resilience.

Second, our modelled estimates show that several priority countries we identified either remain unenrolled in GLASS or report only limited AMR data. ^75^ In Latin America, participation remains sparse, with many countries (e.g., Paraguay, Ecuador, Suriname, Belize) not contributing resistance data, while in sub-Saharan Africa (e.g., Sudan, Tanzania, Burkina Faso), coverage is present but limited in scope and representativeness. ^75^ This pattern indicates that even where GLASS enrolment has occurred, the absence of routine, nationally representative data continues to constrain local and global AMR estimation. Consequently, global AMR estimates continue to be disproportionately shaped by data from better-resourced countries, while high-burden contexts remain largely invisible. ^61^ The limited coverage and incomplete reporting from these regions hamper the accuracy of global AMR estimates, meaning that model-based approaches often provide the comprehensive evidence base for decision-making and prioritisation in such contexts. However, while these models are themselves affected by the same underlying data limitations, they remain essential for generating structured, comparable estimates that would otherwise be unavailable.

By linking estimated prevalence levels to contextual factors such as healthcare utilisation, socioeconomic development, and demographic pressures, the covariate context makes it possible to identify countries where these underlying conditions may be amplifying resistance. Countries such as Bangladesh, Nigeria, and Tanzania exhibit a combination of high population density, rapid urbanisation, and limited WASH infrastructure—conditions that are strongly linked with AMR. For instance, Bangladesh exemplifies how dense populations and poor sanitation foster environmental persistence of resistant enteric bacteria. A study in Dhaka found gram negative resistant organisms widely distributed across hospital and municipal water sources, highlighting the impact of inadequate WASH infrastructure. ^76^A recent gap analysis across Kenya, Tanzania, Uganda, and Zambia highlighted critical shortfalls in AMR surveillance capacity, including scarce laboratory infrastructure, weak regional data sharing, and limited integration across human, animal, and environmental health sectors. These deficiencies collectively create environments that enable the ongoing persistence of resistant pathogens. ^77^ Similarly, in Tanzania, AMR surveillance and antimicrobial stewardship program implementation remain inconsistent across health facilities, with only a minority conducting routine susceptibility testing or prescription audits. ^77, 78^ In Myanmar, efforts to implement hospital-based AMR surveillance are undermined by operational constraints including the absence of trained microbiologists, inadequate digital infrastructure, and inconsistent data collection systems highlighting the depth of health system limitations. ^79^ In Paraguay and Belize, studies indicate persistent difficulties in enforcing prescription-only antimicrobial policies, reflecting stewardship and governance gaps. ^67^ Similarly, in Niger and Burkina Faso, capacity assessments show critically limited laboratory infrastructure and AMR surveillance capabilities across sub-Saharan Africa. ^67^ Together, these examples provide a plausible explanation and may help explain the consistent appearance of certain countries across pathogens in our models.

Also, the clinical relevance of gram-negative pathogens heightens the need for prioritisation. CRAB and 3GCR-EC illustrate different but complementary pressures within gram-negative AMR. CRAB reflects hospital-associated risk, thriving in intensive care settings and device-related infections where infection prevention and diagnostic gaps are critical. ^4^ In contrast, 3GCR-EC represents widespread community and hospital exposure, frequently detected in urinary and bloodstream infections. ^4^ Its prevalence is closely linked to WASH conditions and antibiotic use in primary care. ^4^ A comprehensive approach that includes improved surveillance, infection prevention, and equitable access to new and existing antibiotics is necessary.

Lastly, based on the modelled prevalence levels and temporal dynamics identified in this study, countries can be grouped by distinct surveillance priorities. In high-prevalence settings where resistance is already deeply entrenched—such as Bangladesh, Egypt, Kazakhstan, Ecuador, and Azerbaijan—systematic and continuous surveillance remains critical to track fluctuations within already elevated resistance levels. In contrast, countries showing recent sharp increases, including Tanzania, Niger, Uganda, Vanuatu, Namibia, and Syria, require intensified, time-sensitive surveillance to determine whether these changes reflect temporary variability or sustained escalation. Where resistance continues to rise gradually, as observed in settings such as Nepal, Myanmar, Peru, North Macedonia, and several sub-Saharan African countries (e.g., Kenya, Burkina Faso, Mali), regular time-series surveillance data are needed to verify persistence and detect inflection points in trend. Together, these findings support a tiered surveillance framework: maintaining close observation in established hotspots, strengthening early-warning systems in emerging zones, and ensuring consistent follow-up in countries exhibiting progressive growth.

### Strengths & Limitations

One of the key strengths of this study is the integration of AMR surveillance data ( *ATLAS & ResistanceMap*) spanning 17 years, enabling the assessment of trends across diverse geographical regions. By employing a stacked ensemble modelling framework, we effectively combine multiple machines learning models, improving estimates and reducing the limitations inherent to any single approach. Additionally, incorporating a ST-GPR meta-model further refines our estimates by capturing spatial and temporal correlations while simultaneously quantifying uncertainty. The structured selection of covariates ensures a more nuanced understanding of AMR, extending beyond healthcare metrics to include socio-demographic and environmental factors. Overall, our methodological approach not only improves AMR estimation in data-limited settings but also provides a framework that can be applied to other pathogens of public health importance.

Despite these strengths, several limitations warrant consideration. We derived covariate importance scores, indicating the relative contribution of each covariate to the model’s AMR estimations. However, these importance scores exclusively reflect each covariate’s relative influence within the modelling framework. It inherently lacks the granularity required to infer causal relationships. Although we compiled a comprehensive set of socioeconomic, health, and environmental covariates, data availability varied across countries and years, which may bias results towards better-reported covariates. Additionally, the potential for selection bias must be acknowledged. The laboratory-based AMR data utilized in our analysis, derived from passive surveillance, may result in an over-representation of resistant isolates. This bias is not only methodological but inherent to the way current global AMR surveillance systems are structured, which typically capture samples from individuals who seek care and are more likely to have severe or treatment-refractory infections. In contrast, population-based surveillance, where every eligible case within a defined population is systematically captured, represents a more robust and less biased approach, although it is less implemented due to cost and operational constraints. ^80^ Furthermore, the surveillance data used to train our models predominantly originates from high-income countries, with a considerable deficiency in data from lower middle-income countries. Within these constraints, we attempted to minimise bias through several measures. These included applying strict inclusion criteria such as minimum isolate counts, conducting extensive data-quality checks, harmonising datasets across years and sources, and using modelling approaches that partially pool information across space and time to reduce the influence of outlying or sparsely reported estimates. Despite implementing all feasible measures to mitigate bias, the model is inevitably affected by the inherent limitations of the dataset. Moreover, multi-drug resistance patterns remain outside the scope of this study because such analyses require individual-isolate–level data with linked susceptibility profiles, which are not available in *ResistanceMap.* Although such data are available in isolate-level datasets such as those hosted by Vivli, they are not yet available at the global scale required for comprehensive AMR modelling, representing an important area for future research.

## Conclusion

Our modelled estimates establish a framework to guide surveillance priorities, helping to close data gaps where evidence is most limited. Investment in laboratory capacity, surveillance reporting, and antimicrobial stewardship is essential to translate evidence into action. Expanding surveillance where gaps are greatest will improve global monitoring, enable earlier containment, and support equitable access to treatment.

## Ethics and consent

The study analysed anonymised antimicrobial resistance surveillance data. No individual-level identifiable information was available to the investigators, and no new data were collected. In accordance with national regulations and institutional policies, formal ethical approval was not required.

## Data Availability

The Antimicrobial Testing Leadership and Surveillance (ATLAS) dataset cannot be made available as it is under license by a third party. However, the dataset can be requested from Vivli ( https://amr.vivli.org) using the following information: Data contributor name: Pfizer (AMR); DOI: https://doi.org/10.25934/PR00007930; AMR ID: VIV00007930; and Dataset ID: ATLAS_Antibiotics. Similarly, the data from ResistanceMap can be made available upon request. For correspondence, please contact comms@onehealthtrust.org. One Health Trust. The supplementary appendix is available at: https://doi.org/10.6084/m9.figshare.31077844. ^81^ All code used in the study is available at https://doi.org/10.5281/zenodo.18269992. ^82^ Our study complies with the Guidelines for Accurate and Transparent Health Estimates Reporting (GATHER) recommendations ( *Supplementary Appendix, Table S17*). ^81^
