# Supplementary material for: Prioritization of antimicrobial resistance surveillance of gram-negative pathogens - a stacked modelling approach
Source: Wellcome Open Res. 2026 Feb 20;11:134. [Version 1] doi: 10.12688/wellcomeopenres.25696.1 (PMC13555039; doi:10.12688/wellcomeopenres.25696.1)
Supplement: Peer review history

# Reviewer report: Reviewer response for version 1

**DOI:** 10.21956/wellcomeopenres.28294.r152442

**Published:** 2026-04-06

**Authors:** Gang Wang

**Affiliations:** 1 Université Catholique de Louvain, Brussels, Belgium

This manuscript addresses an important and highly relevant question in global antimicrobial resistance (AMR) surveillance: how to prioritise surveillance strengthening for gram-negative pathogens in countries where routine microbiology capacity is limited. The authors combine AMR data from the Vivli/ATLAS repository and ResistanceMap with 178 country-level covariates and apply a stacked ensemble framework with a spatiotemporal Gaussian process regression meta-model to estimate national AMR prevalence for nine priority pathogen–antibiotic combinations from 2005 to 2021. The policy motivation is clear, the topic is timely, and the work has potential practical value for resource-constrained settings. The manuscript also cites recent and relevant literature, including recent WHO and AMR burden references .

 In its current form, however, I consider several of the evaluation questions best answered as **“Partly”** rather than “Yes”, because some key methodological and interpretive issues remain insufficiently resolved for a paper that aims to guide real-world surveillance prioritisation. The manuscript is generally well written and the modelling pipeline is described at a reasonable level, but important limitations regarding source-data comparability, model transportability, uncertainty-aware prioritisation, and reproducibility should be addressed more explicitly. The study uses over 5.8 million isolates from 82 countries, merges surveillance data with 178 retained covariates after filtering and imputation, excludes country-years with fewer than 30 isolates, and reports out-of-sample performance for most pathogen–drug combinations in the range of R² 0.65–0.93. Those are strengths. However, the same methods section also makes clear that the model is trained on a combined dataset built from heterogeneous surveillance sources and then applied only to countries lacking surveillance, which raises important questions about generalizability and comparability.

 A major concern is that the **validation strategy does not fully match the intended application**. The model is used to generate estimates for countries with no surveillance data, yet performance is assessed using stratified train-test splitting within the observed dataset, plus internal cross-validation and bootstrap resampling. This demonstrates internal predictive skill, but it is not equivalent to testing how well the framework extrapolates to completely unobserved countries or regions. For a surveillance-prioritization tool, this distinction matters greatly in practice. I therefore think the study design is only **partly** sufficient as presented. The authors should either perform a more stringent external-style validation, such as leave-one-country-out, leave-one-region-out, or temporally blocked validation, or explicitly temper their claims about model performance in data-absent settings.

 A second major issue is the **harmonization of different AMR data sources**. ResistanceMap provides aggregated country-level resistance estimates from invasive isolates obtained from blood and cerebrospinal fluid, whereas ATLAS contains isolate-level susceptibility data from multiple specimen types including blood, sputum, urine, abscess material, and wound swabs. The manuscript also reclassifies intermediate susceptibility as resistant to align the datasets. While this harmonization is understandable, it introduces non-trivial comparability concerns, especially for pathogen–drug combinations whose resistance prevalence may vary substantially by specimen type, clinical syndrome, and care setting. In my view, this issue is central rather than peripheral because the study’s outputs are intended to inform surveillance investment decisions. The authors should provide sensitivity analyses using more comparable subsets where feasible, or at least expand the discussion of the likely direction and magnitude of bias introduced by combining these data streams.

 Third, although the statistical framework is sophisticated and in many respects appropriate, the **interpretation of the model outputs and covariate importance is somewhat stronger than the design strictly supports**. The manuscript appropriately states that covariate importance scores do not imply causality, and the results section notes that governance, health-system financing, socioeconomic variables, WASH, agricultural, nutritional, and environmental factors rank highly. However, parts of the Discussion read as though these factors are being interpreted as mechanistic drivers rather than predictive correlates within a modelling framework. This is especially important because policy readers may overread such results as evidence for causal leverage points. I therefore rate the statistical analysis and its interpretation as **partly** appropriate: the modelling itself is broadly reasonable, but the interpretation should be more restrained and more clearly separated into predictive versus causal language.

 Fourth, the manuscript would benefit from a stronger **uncertainty-aware prioritisation framework**. The authors derive three prioritisation indicators: absolute estimated prevalence in 2021, short-term change from 2016 to 2021, and long-term change from 2005 to 2021. These are sensible indicators. However, if the real goal is to decide where surveillance should be expanded first, then uncertainty should play a more direct role in the ranking or categorisation of countries. A country with a high predicted prevalence but very wide uncertainty may warrant a different operational response from a country with similarly high predicted prevalence and tighter uncertainty. Since the meta-model is probabilistic and the manuscript notes that 95% uncertainty intervals were generated, the paper would be considerably stronger if uncertainty were incorporated explicitly into the prioritisation logic rather than being treated mainly as a technical output.

 Fifth, the paper acknowledges important data limitations that, in my view, should have even greater weight in the conclusions. The authors note that passive laboratory surveillance may over-represent resistant isolates, that the surveillance data used to train the models predominantly originate from high-income countries, and that lower-middle-income countries remain underrepresented. These are highly relevant limitations for a manuscript aiming to guide surveillance priorities in LMICs. The model may still be useful as a screening or prioritisation aid, but the conclusions should emphasise more clearly that the estimates are not substitutes for representative population-based surveillance and should not be interpreted as precise prevalence measurements in countries without routine data. On this point, the conclusions are only **partly** supported by the results in their current wording.

 On reproducibility, I also think **“Partly”** is the most accurate answer. The manuscript provides substantial methodological detail, identifies the software environment and packages, notes compliance with GATHER, and makes the analysis code available on Zenodo. Those are clear strengths. However, the core ATLAS dataset is not openly available because it is under third-party license, and ResistanceMap is available only upon request. This means the study is not fully reproducible from openly accessible source data, even though code and supplementary materials are provided. I therefore would not mark “Yes” for full source-data availability or full reproducibility.

 To make the article scientifically stronger, I think the following points should be addressed:

1. The authors should clarify that internal cross-validation performance does not fully establish transportability to countries with no surveillance data, and ideally provide a more demanding validation scheme closer to the intended use case.
2. The authors should justify more fully the harmonisation of ATLAS and ResistanceMap, including the handling of different specimen types and the reclassification of intermediate isolates as resistant, and add sensitivity analyses if feasible.
3. The authors should integrate predictive uncertainty more explicitly into the surveillance-prioritisation framework.
4. The authors should distinguish more clearly between predictive covariate importance and causal inference, and avoid implying mechanism where the modelling design only supports association.
5. The authors should make the practical interpretation of the outputs more cautious, emphasising that these estimates are best used to guide surveillance expansion rather than replace representative surveillance.
6. The authors should clarify the degree of reproducibility that is realistically achievable given the licensed and request-only source datasets.

I also have several more minor but still useful suggestions. The rationale for classifying a country as having surveillance if data were available for at least one year between 2016 and 2021 should be explained more clearly, especially since the modelling period spans 2005–2021. The manuscript would also benefit from a clearer explanation of how policymakers should operationalize the rankings in practice, for example whether they are intended to guide GLASS enrolment, sentinel-site placement, laboratory capacity investment, or prioritisation of specific pathogen–drug combinations. In addition, moving a concise table of the most influential covariates into the main text would improve readability and reduce reliance on the Supplementary Appendix.

 In summary, this is a timely, relevant, and potentially useful study with real policy significance.

 The overall framework is promising, and the manuscript already has several notable strengths. However, because of the issues above, especially those related to external transportability, data-source comparability, uncertainty-aware prioritisation, and interpretation of model-derived covariates, I would currently answer several of the review questions as **“Partly.”** Addressing these points would materially improve both the scientific robustness and the real-world usefulness of the work.

Is the work clearly and accurately presented and does it cite the current literature?

Partly

If applicable, is the statistical analysis and its interpretation appropriate?

Partly

Are all the source data underlying the results available to ensure full reproducibility?

Partly

Is the study design appropriate and is the work technically sound?

Partly

Are the conclusions drawn adequately supported by the results?

Partly

Are sufficient details of methods and analysis provided to allow replication by others?

Partly

Reviewer Expertise:

Antimicrobial resistance, antibacterial drug discovery, host–pathogen interactions, bacterial persistence, infectious disease microbiology, translational antimicrobial research.

I confirm that I have read this submission and believe that I have an appropriate level of expertise to confirm that it is of an acceptable scientific standard, however I have significant reservations, as outlined above.

# Response

**Published:** 2026-06-12

**Authors:** SNEHA KOTIAN

**Affiliations:** Global Health, Amsterdam UMC Locatie AMC, Amsterdam, North Holland, The Netherlands

We thank Reviewer 2 for their careful and constructive assessment of our manuscript. 

 We have revised the manuscript and added Supplementary Appendix 2 to address these points, and we provide a point-by-point response below. 

**Comment no 1:**  A major concern is that the validation strategy does not fully match the intended application. The model is used to generate estimates for countries with no surveillance data, yet performance is assessed using stratified train-test splitting within the observed dataset, plus internal cross-validation and bootstrap resampling. This demonstrates internal predictive skill, but it is not equivalent to testing how well the framework extrapolates to completely unobserved countries or regions. For a surveillance-prioritization tool, this distinction matters greatly in practice. I therefore think the study design is only partly sufficient as presented. The authors should either perform a more stringent external-style validation, such as leave-one-country-out, leave-one-region-out, or temporally blocked validation, or explicitly temper their claims about model performance in data-absent settings.  The authors should clarify that internal cross-validation performance does not fully establish transportability to countries with no surveillance data and ideally provide a more demanding validation scheme closer to the intended use case. 

**Answer no 1: **  We agree that validation should be aligned as closely as possible with the intended application of the model. We also recognise that our original wording did not make the validation design sufficiently clear. As described in the manuscript, we used a stringent validation approach consistent with the reviewer’s recommendation, using stratified country level holdout validation.  Thus, all observations from a given country across all available years were assigned exclusively to either the training or the testing set, with stratification by WHO region and World Bank income group to preserve geographic and socioeconomic representation in both sets. The reported out-of-sample performance therefore reflects estimation for entirely unseen countries within the testing dataset.  To further contextualise estimates in data-absent settings, we also calculated Mahalanobis distance as a supplementary measure of how far countries without surveillance data lay from the covariate space represented in the training data. (Supplementary Appendix 2)   We have revised the manuscript to make the country-level holdout explicit, and added the following text to the methods and discussion section (Lines: 114-120; 158-164; 588-602)     

**Comment no 2: **  A second major issue is the harmonization of different AMR data sources. ResistanceMap provides aggregated country-level resistance estimates from invasive isolates obtained from blood and cerebrospinal fluid, whereas ATLAS contains isolate-level susceptibility data from multiple specimen types including blood, sputum, urine, abscess material, and wound swabs. The manuscript also reclassifies intermediate susceptibility as resistant to align the datasets. While this harmonization is understandable, it introduces non-trivial comparability concerns, especially pathogen–drug combinations whose resistance prevalence may vary substantially by specimen type, clinical syndrome, and care setting. In my view, this issue is central rather than peripheral because the study’s outputs are intended to inform surveillance investment decisions. The authors should provide sensitivity analyses using more comparable subsets where feasible or at least expand the discussion of the likely direction and magnitude of bias introduced by combining these data streams.  The authors should justify more fully the harmonisation of ATLAS and ResistanceMap, including the handling of different specimen types and the reclassification of intermediate isolates as resistant, and add sensitivity analyses if feasible. 

**Answer no 2:**  We agree with the reviewer that harmonising ResistanceMap and ATLAS introduces potential comparability concerns. ResistanceMap provides aggregated country-level resistance estimates from invasive isolates obtained from blood and cerebrospinal fluid, whereas ATLAS includes isolate-level data from a broader range of specimen types, including blood, sputum, urine, abscess material, and wound swabs. We also agree that resistance prevalence may vary by specimen type, clinical syndrome, and care setting, and that this issue is relevant for interpreting outputs intended to guide surveillance prioritisation.  To address this concern, we performed an additional sensitivity analysis (Supplementary Appendix 2) in which ATLAS was restricted to blood and cerebrospinal fluid specimens only, while ResistanceMap was kept unchanged. Intermediate susceptibility results were classified as resistant, consistent with the main analysis. The blood/CSF-restricted ATLAS sensitivity estimates were consistent with the observed resistance estimates used in the main analysis, which combine ResistanceMap and all eligible ATLAS specimens. For carbapenem-resistant A. baumannii and third-generation cephalosporin-resistant E. coli, the Pearson correlation coefficients between the two sets of estimates were 0.992 and 0.968, respectively. These findings suggest that, for the evaluated pathogen–antibiotic combinations and overlapping country-years, restricting ATLAS to invasive specimens did not change the observed resistance estimates significantly.  However, a full blood/CSF-restricted analysis was only partly feasible and would have substantially reduced the usable ATLAS data.(Supplementary Appendix 2) Across the pathogen–antibiotic combinations included in our analysis, only 12.1% to 25.2% of ATLAS isolates were from blood or cerebrospinal fluid; the remaining isolates were from other or unknown specimen types. Restricting ATLAS to blood/CSF would therefore have reduced the number of eligible country-year observations, particularly in already data-sparse settings. In addition, ResistanceMap is available only as an aggregated resistance measure and cannot be further stratified by specimen type, clinical syndrome, or care setting.  These results highlight a trade-off between comparability and coverage. Restricting ATLAS to invasive specimens improves comparability with ResistanceMap but substantially reduces surveillance coverage. Conversely, retaining the broader ATLAS dataset preserves country-year coverage, which is important for a study focused on surveillance gaps and prioritisation. Because ATLAS contributed fewer tested isolates than ResistanceMap, changes to the ATLAS specimen definition had limited influence on the combined observed resistance estimates in the sensitivity analysis   We have added the detailed sensitivity analyses in (Supplementary Appendix 2) for your reference.  

**Comment no 3:**  Third, although the statistical framework is sophisticated and in many respects appropriate, the interpretation of the model outputs and covariate importance is somewhat stronger than the design strictly supports. The manuscript appropriately states that covariate importance scores do not imply causality, and the results section notes that governance, health-system financing, socioeconomic variables, WASH, agricultural, nutritional, and environmental factors rank highly. However, parts of the Discussion read as though these factors are being interpreted as mechanistic drivers rather than predictive correlates within a modelling framework. This is especially important because policy readers may overread such results as evidence for causal leverage points. I therefore rate the statistical analysis and its interpretation as partly appropriate: the modelling itself is broadly reasonable, but the interpretation should be more restrained and more clearly separated into predictive versus causal language.  The authors should distinguish more clearly between predictive covariate importance and causal inference, and avoid implying mechanism where the modelling design only supports association. 

**Answer no 3:**  We agree with the reviewer that correlation does not imply causality and realise we might not have made this distinction clear enough throughout the manuscript. We have revised the manuscript in response to this suggestion. (Lines: 432-435) We removed text that could have been interpreted as implying causal mechanisms. (Lines: 482-483) 

**Comment no 4:**  Fourth, the manuscript would benefit from a stronger uncertainty-aware prioritisation framework. The authors derive three prioritisation indicators: absolute estimated prevalence in 2021, short-term change from 2016 to 2021, and long-term change from 2005 to 2021. These are sensible indicators. However, if the real goal is to decide where surveillance should be expanded first, then uncertainty should play a more direct role in the ranking or categorisation of countries. A country with a high predicted prevalence but very wide uncertainty may warrant a different operational response from a country with similarly high predicted prevalence and tighter uncertainty. Since the meta-model is probabilistic and the manuscript notes that 95% uncertainty intervals were generated, the paper would be considerably stronger if uncertainty were incorporated explicitly into the prioritisation logic rather than being treated mainly as a technical output.  The authors should integrate predictive uncertainty more explicitly into the surveillance-prioritisation framework. 

**Answer no 4:**  We agree that uncertainty should be incorporated more directly into the interpretation of surveillance priorities, rather than presented only as a technical model output. In response, we added a rank-stability analysis to assess how robust country prioritisation rankings were to predictive uncertainty. This analysis propagated uncertainty by repeatedly sampling plausible resistance values from the estimated uncertainty intervals and re-ranking countries across 1,000 simulations. Countries were then classified as having high, moderate, or low rank stability based on the probability of remaining in the same rank quintile as the point estimate. (Supplementary Appendix 2)  The results showed that prioritisation rankings were generally robust to predictive uncertainty. For carbapenem-resistant A. baumannii, 55.2% of country-year estimates showed high rank stability, 41.3% moderate stability, and 3.4% low stability. For third-generation cephalosporin-resistant E. coli, 83.0% of country-year estimates were classified as high stability, 15.5% as moderate stability, and 1.5% as low stability. In 2021, the highest-ranked countries for both pathogen–antibiotic combinations were generally classified as high stability.  

**Comment no 5:**  Fifth, the paper acknowledges important data limitations that, in my view, should have even greater weight in the conclusions. The authors note that passive laboratory surveillance may over-represent resistant isolates, that the surveillance data used to train the models predominantly originate from high-income countries, and that lower-middle-income countries remain underrepresented. These are highly relevant limitations for a manuscript aiming to guide surveillance priorities in LMICs. The model may still be useful as a screening or prioritisation aid, but the conclusions should emphasise more clearly that the estimates are not substitutes for representative population-based surveillance and should not be interpreted as precise prevalence measurements in countries without routine data. On this point, the conclusions are only partly supported by the results in their current wording.  The authors should make the practical interpretation of the outputs more cautious, emphasising that these estimates are best used to guide surveillance expansion rather than replace representative surveillance. 

**Answer no 5:**  We agree with the reviewer that these limitations should be given appropriate weight in the interpretation of the study findings. We have revised the conclusion. (Lines: 613-622)  At the same time, we would like to clarify that passive laboratory-based surveillance may over-represent resistant isolates, but this does not preclude its use for prioritising surveillance strengthening. Rather, it highlights the need to interpret the outputs in relation to the surveillance system they are intended to support. Our estimates are most appropriately viewed as tools to identify where laboratory surveillance capacity, coverage, and reporting could be expanded or strengthened, while recognising that population-based surveillance systems would provide more precise estimates of resistance prevalence. To further support interpretation in countries without observed AMR data, we now also refer to the Mahalanobis distance analysis. This analysis assesses how similar countries without surveillance data are to countries represented in the training dataset. Countries with larger Mahalanobis distances are less well represented within the observed covariate space, indicating that predictions for these settings involve greater extrapolation from the training data. These countries may therefore be important targets for future surveillance expansion, both to strengthen local evidence generation and to improve the empirical basis for future modelled estimates.  

**Comment no 6:**  On reproducibility, I also think “Partly” is the most accurate answer. The manuscript provides substantial methodological detail, identifies the software environment and packages, notes compliance with GATHER, and makes the analysis code available on Zenodo. Those are clear strengths. However, the core ATLAS dataset is not openly available because it is under third-party license, and ResistanceMap is available only upon request. This means the study is not fully reproducible from openly accessible source data, even though code and supplementary materials are provided. I therefore would not mark “Yes” for full source-data availability or full reproducibility.  The authors should clarify the degree of reproducibility that is realistically achievable given the licensed and request-only source datasets 

**Answer no 6:**  We agree that the study cannot be reproduced from openly downloadable AMR source data alone as the underlying AMR datasets are governed by third-party access conditions. The ATLAS dataset is subject to licence restrictions and cannot be redistributed by the authors, and ResistanceMap data are available upon request from One Health Trust. However, we would respectfully clarify that this reflects the governance conditions of the source datasets rather than a limitation in the transparency of the analytical workflow. This approach is common for studies using licensed or request-access health surveillance datasets, where authors are not permitted to re-host the original data but can provide sufficient information for other researchers to request access through the appropriate mechanisms. To support reproducibility as fully as possible within these constraints, we have provided the relevant details and access routes for both AMR data sources. Researchers who obtain access to the ATLAS and ResistanceMap datasets through these mechanisms should therefore be able to reproduce the analytical workflow and model outputs. We have revised the data availability statement to make this distinction clear. (Lines 639-640)  We thank the reviewer again for these helpful comments. The revisions have strengthened the manuscript.

# Reviewer report: Reviewer response for version 1

**DOI:** 10.21956/wellcomeopenres.28294.r149236

**Published:** 2026-03-26

**Authors:** Chloé Smit

**Affiliations:** 1 University of Technology Sydney, Broadway, New South Wales, Australia

 This article presents important and timely work that substantially contributes to global antimicrobial resistance (AMR) surveillance efforts. The authors address a critical gap by explicitly considering surveillance priorities in low‑ and middle‑income countries (LMICs), where data limitations often constrain decision‑making despite a high AMR burden. The methodological approach is robust, drawing on large epidemiological datasets, and the analytical framework is appropriate and well justified. The findings have clear practical relevance for shaping global AMR surveillance policy.

**Strengths**

- **Significant contribution to surveillance priorities:** By integrating LMIC contexts, the study supports more equitable global surveillance planning.
- **Robust methodology:** The use of extensive epidemiological data and sound analytical techniques strengthens the validity of the findings.
- **Policy relevance:** The study delivers insights that can assist decision‑makers in directing surveillance resources more effectively.

**Areas for Minor Revision**

1. **Include covariate results in the main text (not only in Supplementary Materials).**
  The manuscript currently directs readers to supplementary tables for the results of the 20 covariates. Presenting at least a S3 and S4 summary tables in the Results section would greatly enhance readability and coherence, reducing the need for readers to switch between documents.
2. **Clarify which covariate findings originate from your analysis versus those from the wider literature.**
  Some covariates such as chronic disease factors (e.g., obesity, diabetes) and agricultural variables (e.g., cropland use, aquaculture production) are discussed in greater detail in the Discussion than in the Results. As written, it is sometimes unclear whether these points reflect your own findings or external sources.
  Explicitly presenting these covariate results in the Results section will help avoid ambiguity and strengthen the internal logic of the manuscript.
3. **Consider referencing relevant One Health AMR literature to contextualise the covariate findings in the discussion.**
  The authors may wish to consider referencing our recent umbrella review, *One Health Determinants of *Escherichia coli* Antimicrobial Resistance in Humans in the Community: An Umbrella Review*, which discusses how the relevance and direction of several covariates (including chronic disease prevalence and agricultural factors) vary substantially across country contexts. Incorporating this reference may help frame your findings of *Escherichia coli *within the broader one-health frame and further emphasise the country‑dependent nature of these determinants.

**Conclusion**

 This is a valuable and well‑executed study that provides meaningful insights for global AMR surveillance, particularly in LMIC contexts. Only minor clarifications are needed to improve the presentation and interpretation of the covariate analyses. Once these points are addressed, the manuscript will be even clearer and easier for readers to follow.

Is the work clearly and accurately presented and does it cite the current literature?

Yes

If applicable, is the statistical analysis and its interpretation appropriate?

I cannot comment. A qualified statistician is required.

Are all the source data underlying the results available to ensure full reproducibility?

Yes

Is the study design appropriate and is the work technically sound?

Yes

Are the conclusions drawn adequately supported by the results?

Yes

Are sufficient details of methods and analysis provided to allow replication by others?

Yes

Reviewer Expertise:

pharmacoepidemiology, geospatial analysis, Escherichia coli antimicrobial resistance

I confirm that I have read this submission and believe that I have an appropriate level of expertise to confirm that it is of an acceptable scientific standard.
